# Using volunteers to improve access to community rehabilitation in palliative care: the St Christopher's Living Well at Home Team

**DOI:** 10.3389/fresc.2023.1229442

**Published:** 2023-09-18

**Authors:** Gail Preston, Sanketh Rampes, Joanne Bayly, Helena Talbot Rice, Ralitsa Angelova, Heather Richardson, Matthew Maddocks

**Affiliations:** ^1^ St Christopher’s Hospice, London, United Kingdom; ^2^ Faculty of Life Sciences and Medicine, King's College London, London, United Kingdom; ^3^ King’s College London, Cicely Saunders Institute, London, United Kingdom; ^4^ St Barnabas Hospice, Worthing, United Kingdom

**Keywords:** rehabilitation, palliative care, hospice care, volunteers, community health services

## Abstract

**Background:**

UK hospices often provide outpatient rehabilitation services for people with advanced progressive illness. However, some people are unable to travel, leading to inequity in rehabilitation access.

**Objectives:**

The Living Well at Home Team (LWAHT) at St Christopher's Hospice aimed to evaluate whether using volunteers to support rehabilitation in peoples’ homes improved the reach of rehabilitation for people living in underserved localities and if it supported people to optimise their functional independence.

**Methods:**

This service improvement project evaluated hospice rehabilitation uptake during the implementation of volunteer-supported community rehabilitation. Following assessment by an LWAHT therapist, eligible people were matched with a trained volunteer who supported four to eight rehabilitation sessions in the person's home. The evaluation assessed uptake of the rehabilitation sessions. Mobility, wellbeing, and goal attainment outcomes were assessed by the Life-Space Assessment (LSA), General Health Questionnaire (GHQ), and Goal Attainment Scale (GAS), respectively.

**Results:**

In the first year, 183 patients were referred to the LWAHT; 123 were assessed and 96 received rehabilitation including 56 who were matched with a volunteer. Following volunteer support, patients reported significant improvements in mobility [LSA median 20 (IQR, 3.5–27.8)], general health [GHQ −2 (−5.25 to 0)], and achievement of goals [GAS T-score +8 (0–18.4)].

**Conclusions:**

It was feasible to support community rehabilitation using hospice volunteers for people with advanced progressive illness. The LWAHT service also increased the uptake of hospice centre-based rehabilitation. Further work should test efficacy and identify patients requiring additional professional input.

**Key message:**

This is the first known study reporting on the use of trained rehabilitation volunteers to extend the reach of hospice rehabilitation services. People with limited access to the hospice, because of geographical location or personal circumstances, valued and benefited from tailored rehabilitation supported by the volunteers in their own homes.

## Introduction

People with advanced progressive conditions often prioritise functional independence and maintaining usual roles and routines for as long as possible (1–4). A high symptom burden often leads to functional loss (5) and disability in activities of daily living (ADL), especially in the last 6 months of life. Functional decline drives hospice admission, and functional needs relating to this decline can be addressed via palliative rehabilitation, prior to and/or during admission (6–8). While many UK hospices provide rehabilitation services, there is wide variation in rehabilitation workforce capacity, organisation, and interventions provided (9, 10).

St Christopher's Hospice, a charitable trust in southeast London, provides specialist palliative care and support to over 5,000 adults each year. People referred to the hospice have access to rehabilitation and wellbeing services, including gym classes (11) and function oriented care (12). However, evaluation of hospice referrals and activity identified that many people in the community who are eligible for referral are unable to access rehabilitation services at the hospice (due to geographical, physical, social, or emotional constraints), despite having functional goals and rehabilitation needs.

Expanding services to a larger cohort of people living in the community would increase reach and access to rehabilitation, but requires additional staffing resource. There is early evidence that volunteers can support rehabilitation delivered in the home setting (12). Volunteers may help patients master rehabilitation interventions and embed them into their day-to-day realities.

This service development project aimed to expand the provision of rehabilitation to people not able to access hospice outpatient services. It modelled a rehabilitative palliative approach using innovative roles for volunteers, to reach people in their home environment who would otherwise be unable to access rehabilitation. This provides the rationale for the working practices, service evaluation processes, and outcomes data.

## Methods

We followed The Model for Service Improvement (13). Following advice from the UK Health Research Authority, the study gained approval from the Hospice Trust R&I lead and Caldicott Guardian. The Living Well at Home Team (LWAHT) service is reported following the TIDieR checklist (14) and the Squire reporting guidelines (15).

The LWAHT aimed to optimise function and wellbeing by providing a practical rehabilitation service in the homes for people unable to access the hospice buildings. The objectives were to enable patients and carers to achieve personal goals, self-manage symptoms using non-pharmacological strategies, and remain as independent as possible within the limitations of illness.

### Living well at home team

The LWAHT consisted of a Team Lead (Band 6 specialist physiotherapist), 0.4 full-time equivalent (FTE), and a Senior Rehabilitation Assistant Practitioner (Band 4-trained support worker), 0.8 FTE. Hospice volunteer and finance services supported recruitment, training of volunteers, and reimbursement of travel expenses. Volunteers were recruited from the existing cohort of patient-facing volunteers and had completed a comprehensive patient-facing training programme. This included scope of role and responsibilities, confidentiality, infection control, and skills for communicating with patients and family members. Additional training specific to the LWAHT programme included training in the rehabilitation intervention components, including supporting and progressing exercises, strategies to support mobility and functioning in activities of daily living, use of walking aids, and non-pharmacological symptom self-management strategies for breathlessness and pain. Training ensured volunteers could conduct a basic risk assessment at the start of each session (see Supplementary Material). This was to ensure people were well enough to engage in rehabilitation that day and to identify if new concerns had arisen that needed communicating to the clinical team. Volunteers had access to clinical staff throughout the visit and followed a lone working protocol involving communication procedures with the base team at the start and end of visit. Clinical staff monitored volunteers’ written reports following each session and entered the details into the person's medical record. Volunteers were encouraged to attend monthly clinical and peer supervision and support sessions.

### Patient eligibility and referral

Patients under the current care of St Christopher's could be referred to the LWAHT. Members of the multi-disciplinary team (MDT) were encouraged to refer people, through the existing Electronic Patient Record system, if they had a rehabilitation goal and were unable to attend outpatient services. Each referral was reviewed and, if accepted, the physiotherapist contacted patients via telephone to assess their understanding of the service, functional goals, and home environment and potential risks.

### LWAHT volunteer rehabilitation programme

For suitable patients, a home visit was arranged during which an LWAHT physiotherapist completed a comprehensive assessment and devised a collaborative rehabilitation plan based on agreed goals (including relatives and/or carers when the patient desired). As indicated, the physiotherapist addressed immediate concerns and short-term goals during the first visit, referred to another rehabilitation service, and/or advised matching with a volunteer.

The LWAHT rehabilitation assistant matched patients with a volunteer, considering availability and ease of travel. Once matched, a joint home visit with the volunteer and a member of the LWAHT was arranged to discuss the patient's goal(s) and rehabilitation plan. Components of the plan to be supported during volunteer visits, including any exercises, techniques, or advice, were practised and clarified.

**Figure 1:**
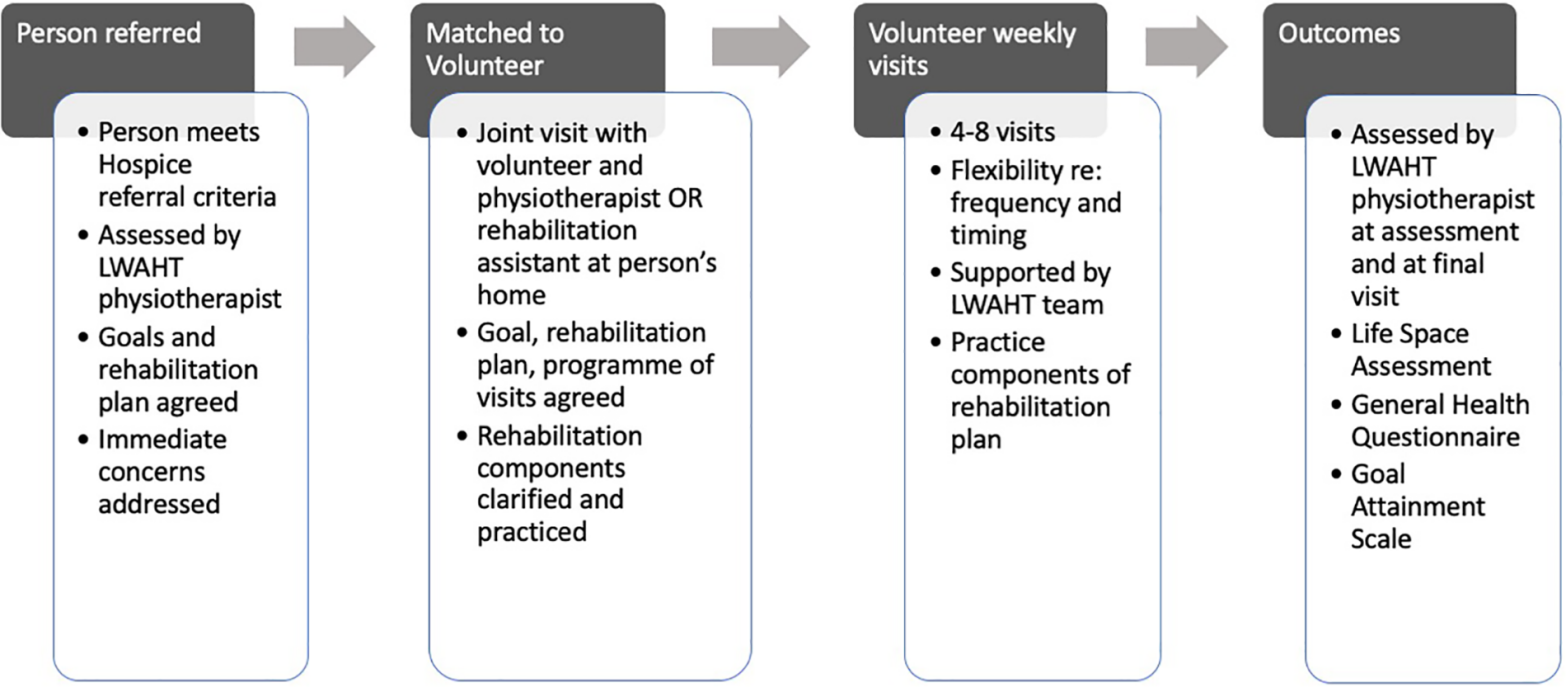
Intervention flow.

The programme typically involved four to eight visits on a weekly basis, with flexibility regarding visit frequency and intervals, to accommodate patient needs and preferences (Figure 1). Appointments were made on agreed times and days, during Monday to Friday working hours of the LWAHT. Volunteers contacted patients on the morning of each planned visit to check if the patient was expecting the visit. A visit report was written and submitted by the volunteer within 48 h and scanned to the patient record.

The volunteer worked with the patient to complete the rehabilitation programme, monitoring the patient throughout. If concerns arose, they were advised to stop the session and to contact the LWAHT physiotherapist, following the lone working policy.

Following completion of the agreed number of visits, or sooner if the goal was achieved early, the volunteer and physiotherapist visited the patient together to review outcomes.

### Data collection and outcomes

Routine hospice data were collected according to usual hospice practice, including Phase of Illness (16), Australian Karnofsky Performance Status (17) (AKPS), and KATZ Independence in Daily Living Scale (18). Additional outcomes measures, recorded at the first and final visit, assessed mobility, wellbeing, and goal achievement assessed using the Life-Space Assessment (LSA, 0–120, higher scores indicate better mobility) (19), General Health Questionnaire (GHQ, 0–36, higher scores indicate worse wellbeing) (20), and the Goal Attainment Scale (GAS), respectively (Supplementary Material 2) (21, 22). Satisfaction with the programme was using items from the FACIT-Treatment Satisfaction-Patient Satisfaction (23) (FACIT TS-PS) measure.

### Analysis

Participant baseline clinical and demographic characteristics were summarised using descriptive statistics. Paired data for the LSA, GHQ, and GAS were analysed using Student’s *t*-test or Wilcoxon signed-rank test as appropriate. *P*-values <0.05 were deemed statistically significant.

## Results

**Figure 2:**
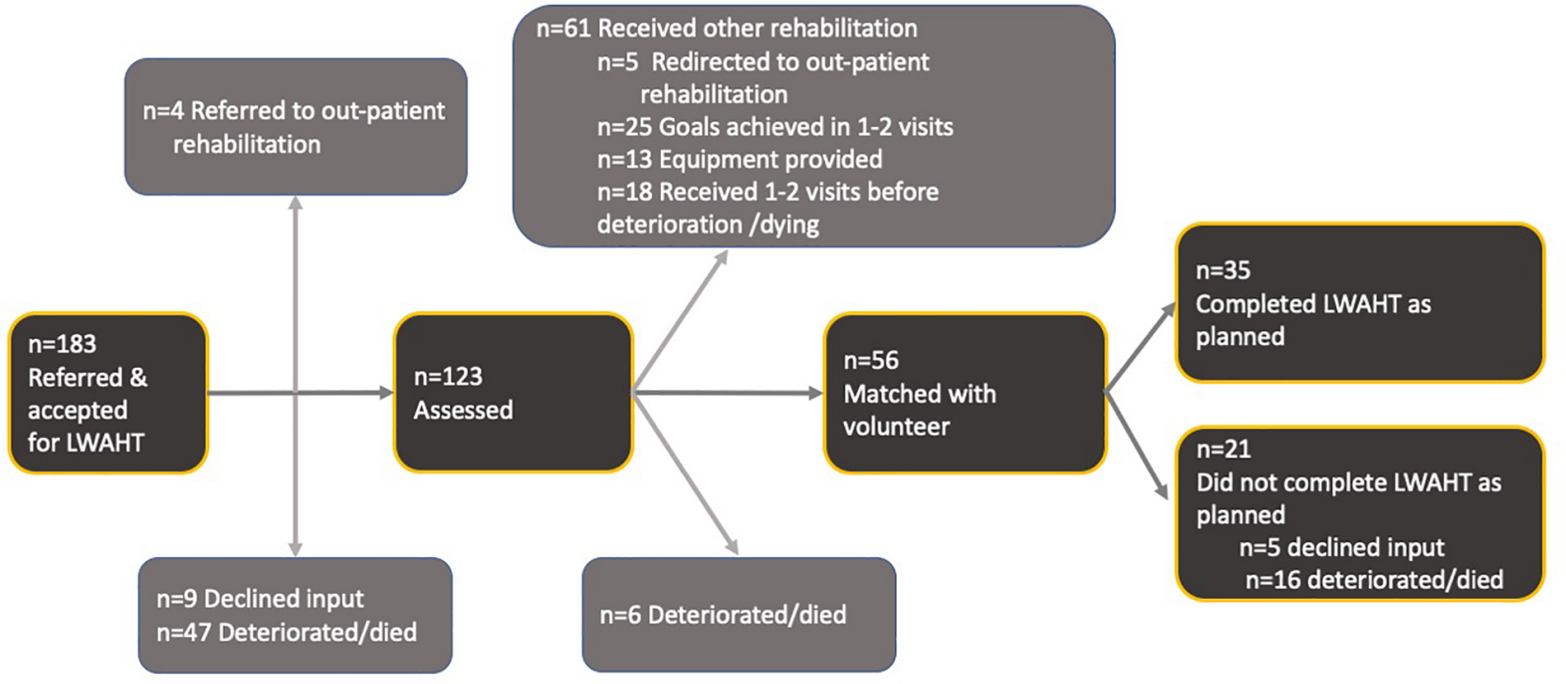
Patient flow through the Living Well at Home Team service.

Over 1 year from January 2017, 1,189 referrals were made to the rehabilitation services. Of these, 183 (16%) people were referred to the LWAHT service (Figure 2). Most people were older (mean age 72 years, SD 16), and two-third had a cancer diagnosis. Chronic obstructive pulmonary disease (COPD) was the most common non-cancer diagnosis. The majority were White British, with approximately one-fifth having a Black or other minority ethnicity (Table 1).

**Table 1:** Clinical and demographic characteristics of people assessed by the LWAHT.

Clinical and demographic and characteristics	Assessed following acceptance of referral (*N* = 123)	Completed LWAHT as planned (*N* = 35)
Mean (SD), years (*N* = 122)	72 (16)	77 (20)
Primary diagnosis cancer, *n* (%)	82 (66)	16 (46)
Bowel	21 (16.9)	5 (14)
Lung	15 (12)	1 (3)
Breast	9 (7)	5 (14)
Prostate	9 (7)	1 (3)
Brain	6 (5)	0
Gynaecological	6 (5)	1 (3)
Haematological	4 (3)	0
Skin	4 (3)	0
Pancreas	2 (2)	1 (3)
Testicular	1 (1)	0
Bladder	1 (1)	0
Liver	1 (1)	0
Other	3 (2)	2 (6)
Primary diagnosis non-cancer, *n* (%)		
COPD	14 (11)	6 (17)
Chronic heart failure	7 (6)	4 (11)
Neurological	8 (7)	1 (3)
Interstitial lung disease	5 (4)	4 (11)
Renal failure	3 (2)	1 (3)
Old age	3 (2)	3 (9)
Liver failure	1 (1)	0
Ethnicity (*N* = 122)		
Afro-Caribbean	15 (12)	2 (6)
Indian	4 (3)	3 (9)
Mixed ethnicity	2 (2)	1 (3)
Other Asian	2 (2)	2 (6)
Other white	13 (11)	2 (6)
White British	83 (71)	23 (66)
Social Status		
Lives alone	42 (34)	18 (51)
Lives with others	82 (66)	17 (49)
Phase of illness (*N* = 93)		
Stable	41 (44)	24 (69)
Unstable	18 (19)	1 (3)
Deteriorating	34 (37)	9 (26)
AKPS^a^ (*N* = 95)		
≤40%	28 (29)	2 (6)
50%	32 (34)	12 (34)
≥60%	35 (27)	11 (46)
KATZ index of independence in ADL^b^ (*N* = 66)		
0–2	14 (8)	4 (11)
3–4	20 (11)	11 (31)
5–6	32 (17)	10 (29)

^a^ AKPS 0%–100%: 0 = dead, 100 = Normal, no complaints, no evidence of disease.

^b^ KATZ, 1 = high, person independent, 0 = low, person very dependent.

Of the 183 people preferred to the LWAHT programme, four (2%) were redirected for outpatient rehabilitation at the hospice site, nine (5%) declined rehabilitation services, and 47 (26%) deteriorated or died prior to the physiotherapist assessment.

Of the 123 who were assessed, 56 (45%) were matched with a volunteer and 35 completed the LWAHT programme in full. Deterioration in condition prevented 16 people from completing the programme and five did not complete as they declined further input.

Sixty-one patients did not require matching with a volunteer. Five (4%) were redirected to outpatient rehabilitation services, 25 (20%) achieved their goals within one to two visits with the LWAHT physiotherapist and/or rehabilitation assistant, and 13 (10%) did not need matching with a volunteer following provision of equipment to support mobility and/or activities of daily living. A further 18 patients received one to two sessions of rehabilitation with the LWAHT but deteriorated or died before being matched with a volunteer.

Following referral to the LWAHT, patients accessing rehabilitation identified goals corresponding with second-level domains of the WHO-International Classification of Function Disability and Health (WHO-ICF) (24). Goals predominantly related to improving functioning in mobility and to improving confidence and reducing fears and anxieties relating to functioning. Other goals related to improving a range of general, community and social, self-care, and domestic activities. Some patients also set goals relating to managing symptoms such as breathlessness and pain, muscle weakness, and balance. Examples of goals are provided in Table 2.

**Table 2:** Example of patient goals by WHO-ICF domain.

Mobility	To get about and walk more easily	To reduce risk of falling	To get to the end of the road
	To get downstairs	To be able to get out of bed	To stand up by myself
Mental functions	To be less anxious and afraid of falling	To feel less lonely	To be more confident in walking
Community social and civic life	To get back to the gym	To grow plants and work in my greenhouse	To go to church
Self-care	To go to the toilet on my own	To have a bath by myself	To put on my own shoes and take them off
Domestic life	To cook and help my wife with housework	To go out shopping	To take the dog for a walk
Muscle functions	To be stronger	To get fitter	To gain some muscle mass, get my arms and legs stronger
Respiratory functions	To be able to manage my breathlessness better	To be more in control of my breathing	To get breathing to be better
Sensory functions	To reduce knee pain	To improve balance	To make neck more comfortable
Relationships	To have more time with family	To be less of a burden on partner	To visit my partner

### Outcomes for patients matched with a volunteer

**Figure 3:**
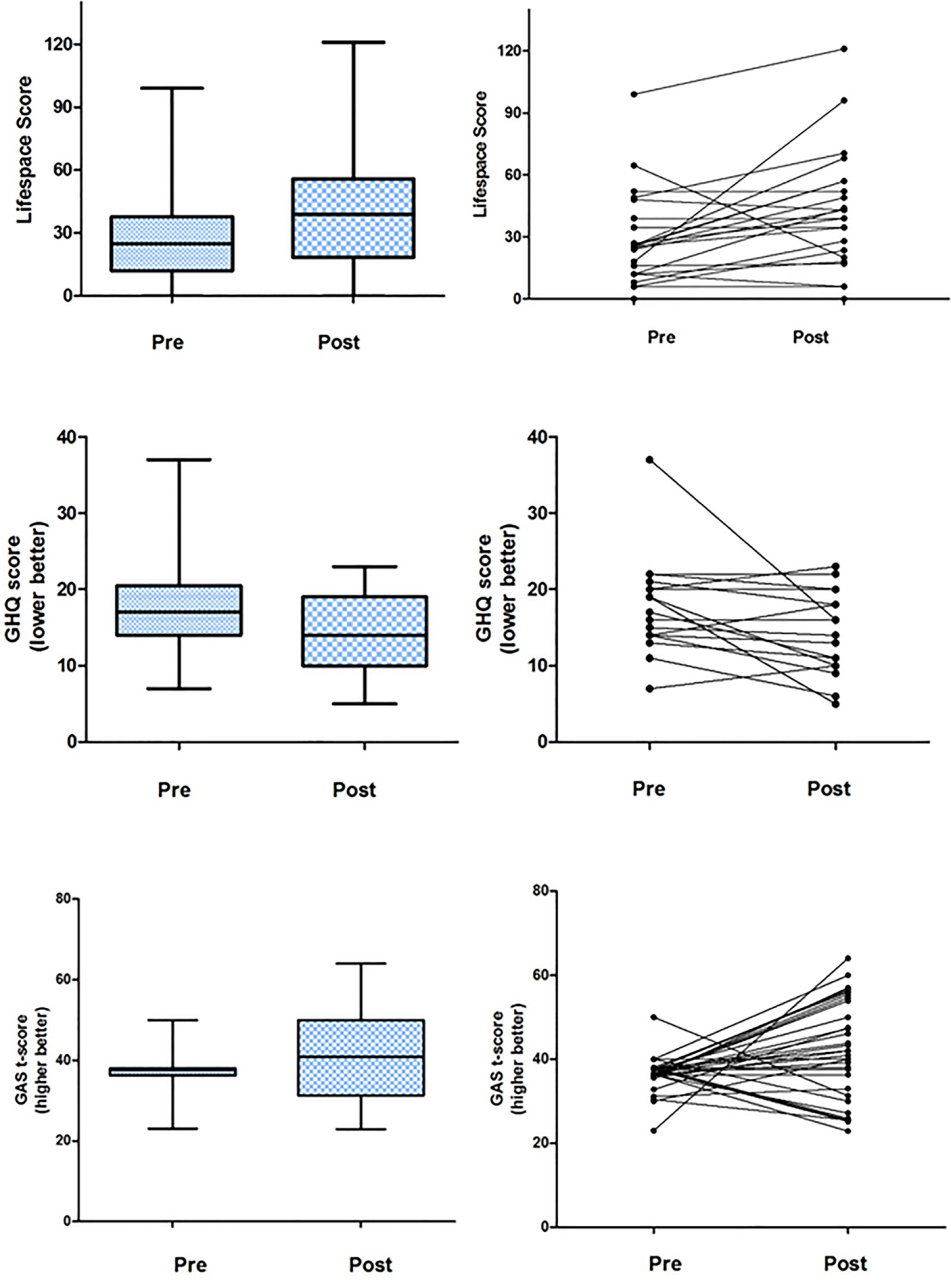
Outcome data for patients matched with a volunteer.

Thirty-five patients completed the LWAHT programme. Paired LSA, GHQ, and GAS were obtained for 19, 20, and 32 patients, respectively. Significant improvements were seen with a median increase of 20 (IQR, 3.5–27.75) in the LSA (*W* = 16, *p* < 0.02), median decrease of −2 (IQR, −5.25 to 0) in GHQ (W = 27, *p* < 0.02), and increases in GAS T-score with a median increase of 8.2 (IQR, 0–18.375) (W = 23, *p* < 0.001). Group and individual data are shown in Figure 3.

### Patient feedback

Patients matched with a volunteer who provided feedback (*n* = 18) reported that the LWAHT therapist and volunteer gave good explanations and that they understood what was important to them. All respondents strongly agreed that they “had confidence in [their] physiotherapist and/or volunteer” and 16/18 “would strongly recommend the service to others” and “would choose to participate in the LWAHT programme again.” Most patients felt that they could say the things that were important to them to the LWAHT staff; however, 2/18 selected “yes, but not as much as I wanted.”

## Discussion

People with reduced functional capacity and geographical and transport barriers had limited access to rehabilitation provided by St Christopher's Hospice, London. A service development project was planned, delivered, and evaluated to improve access to rehabilitation for people living in underserved localities. A volunteer-led rehabilitation outreach programme was designed for people with advanced progressive illness, to be delivered in their own homes and communities. The findings demonstrate that people not previously known to the hospice had increased access to rehabilitation services following referral to the LWAHT programme. While some people's needs were met with one or two contacts with LWAHT clinicians or referral to existing rehabilitation services, just under one-third of those referred were matched with a volunteer, and of these, more than half completed the programme. People who completed the programme showed an improvement in mobility, wellbeing, and made progress towards achieving their goals as measured by LSA, GHQ-12, and GAS, respectively. Feedback from patients was excellent and all recommended the programme to others.

This study is novel for two reasons. First, it is the first known study to report on the use of hospice volunteers to support home rehabilitation to patients receiving care from a hospice. Second, this study demonstrates the efficacy of extending rehabilitation services into the home for people with advanced progressive disease unable to access outpatient rehabilitation services.

In England, two-thirds of hospices are managed in the voluntary sector. Hospices receive between 20% and 50% of total income from statutory funding, relying on charitable donations to cover the remaining costs (25). Volunteers are crucial to the everyday running of a hospice and take a range of roles from patient-facing roles to administration and fundraising. Hospice patient-facing volunteers traditionally do not participate in the physical care of people or the delivery of rehabilitation services. These roles are restricted to healthcare professionals including therapists, nurses, and nursing assistants. This study identified two key benefits. First, providing community rehabilitation by volunteers is cost-effective and sustainable. Volunteers provide an expenses-only service, and the cost of training volunteers for the LWAHT programme is minimal. The cost for the programme was modest, including a small team of an LWAHT Lead (Band 6), 0.4 FTE for 12 months and 0.2 FTE for the following 6 months, and a rehabilitation assistant (Band 4) 0.8 FTE for 16 months, the duration of the evaluation period. Second, giving volunteers a more active role in supporting patients through directly delivering rehabilitation services to people offers them a greater responsibility and sense of fulfilment (26).

People enrolled on the LWAHT programme were unable to access hospice outpatient rehabilitation services due to practical, emotional, or financial reasons, and most were housebound. Community rehabilitation provided by local services has long waiting times, which are incompatible with having an advanced progressive illness. The LWAHT programme provides a model for the provision of rehabilitation for housebound people living in the community, without significant extra cost. In addition, this approach is in line with peoples’ preference for home-based rehabilitation and exercise programmes (27–29).

When evaluating feasibility, consideration of clinical service flow is paramount. This study reports that of 183 people referred to the service, around two-third were assessed. Of these, just under half were accepted onto the LWAHT programme and matched with a volunteer. However, this number does not fully capture the cohort of patients who benefited from the LWAHT service. A proportion of people who were not matched had their goals achieved within one or two visits, or had equipment delivered to them before they deteriorated or died. Therefore, around three-quarters of people who were assessed received rehabilitation. Unlike exercise interventions such as pulmonary rehabilitation for COPD, where completion of the full 8 weeks has strong evidence as a meaningful marker of benefit derived (30), the LWAHT programme does not have an equivalent evidence base for making “completion” a requisite for benefit. People who received input from the rehabilitation service may have benefitted from the service without completion of the volunteer-led programme. For example, one or two sessions with the physiotherapist, the provision of a walking aid, and one or two practice sessions with a volunteer may be sufficient to improve independent physical mobility and health-related quality of life.

The service development and evaluation demonstrate that the LWAHT extended the reach of the hospice rehabilitation services. Tailored rehabilitation is now provided to patients where geographical location or personal circumstances limited access to existing rehabilitation at the hospice.

Lessons were learned from this service development and evaluation project. Collecting outcomes data in addition to routinely collected assessment data was challenging. LSA, GHQ, and GAS were incomplete for some patients who completed the LWAHT programme. The novel use of volunteers rather than qualified clinicians raises questions regarding the quality of the rehabilitation delivered. The results from this study suggest that volunteers under close supervision of clinicians can support effective rehabilitation to this cohort of patients. Given the complexity of patient care and outcomes, regular communication to create an in-depth understanding between clinicians and volunteers is paramount. Further research and evaluation should be conducted to help determine which rehabilitation tasks can be delivered by volunteers and which patients may require rehabilitation from paid healthcare professionals to achieve benefit.

The service was planned and evaluated prior to the COVID-19 pandemic and was sustained after the evaluation with ongoing involvement of 18 volunteers. The COVID-19 pandemic impacted the delivery of rehabilitation in palliative care setting (31) and the involvement of volunteers (32). At the onset of the COVID-19 pandemic, the service moved online and LWAHT volunteers supported people to use the online processes. Face to face visits in the home recommenced once Personal Protective Equipment and vaccinations became available. Working with a population with deteriorating and unpredictable illness trajectories created challenges when matching patients with volunteers. Some were matched but did not remain well enough to benefit extensively from the input. There were also geographical challenges. Volunteers with an established relationship with the hospice who were prepared to work clinically often lived closer to the hospice sites. Finding volunteers who would support patients living at greater distances was more difficult, yet these were often the people who needed the LWAHT service the most as they experienced the most travel issues getting to the hospice for rehabilitation. There was some initial concern that rehabilitation assistants would think they were being replaced by volunteers, but once the role of the volunteer was clearly described, they were reassured and supportive. Other colleagues across the organisation were supportive as the new service represented the hospice value of being “of and for the community.” By increasing connections with the community through volunteers and by valuing their skills, we built relationships. Volunteer satisfaction with their role in the LWAHT was high. Most were retained through the pandemic and have continued to volunteer in the LWAHT since it began 5 years ago.

## Conclusion

In conclusion, this study demonstrates the feasibility and efficacy of using volunteers to support community rehabilitation to patients with advanced progressive illness who were unable to access hospice outpatient services. The LWAHT adds value as part of a broader rehabilitation offering. Following initial assessment and during ongoing access to support, people can choose to receive care from the hospice rehabilitation service that best suits their needs and preferences.

Further research investigating volunteer-delivered home-based rehabilitation in patients with advanced progressive illness is required, specifically validating the efficacy of volunteers and identifying the limitations of using volunteers compared to paid professionals in certain groups of patients.

## Data Availability

The raw data supporting the conclusions of this article will be made available by the authors.
