# Supplementary material for: Using volunteers to improve access to community rehabilitation in palliative care: the St Christopher's Living Well at Home Team
Source: Front Rehabil Sci. 2023 Sep 18;4:1229442. doi: 10.3389/fresc.2023.1229442 (PMC10544964; doi:10.3389/fresc.2023.1229442)
Supplement: Supplementary file 1 [file Presentation1.pdf]

Using volunteers to deliver community rehabilitation in palliative care: The St Christopher's Living Well at Home Team

Supplementary material

1. Outline of the initial Volunteer Training Programme

10:00	**Introductions and Welcomes**
10:15	**Recapping of General Volunteer training:** What did you learn? What questions do you have?
10:30	**Procedures:**<br>• How does the service work?<br>• Procedures for being in touch<br>• Outlook email<br>• Support sessions<br>• Procedures for matching visits and risk<br>• What does a visit look like?<br>• What are your warning signs?<br>• Questions
11:00	Coffee
1110-1300	**Practical Sessions:**<br>• IT Training and documentation<br>• Communication skills<br>• Breathlessness workshop<br>• Interactive activity exploring scope and boundaries of a Living Well at Home Project Volunteer
1300	Lunch
1330	**What is a rehabilitation goal action plan?**
1430	Rehabilitative language<br>Basic moving and handling principals
1500	Mobility – walking aids and exercises
1430	Activities of Daily Living
1600	Practical role plays
1630	Resources, support systems and structures
1700	Questions

2. Clinical Outcome Measures

The University of Alabama at Birmingham (UAB) Study of Aging Life-Space Assessment (LSA)[1] was used to measure mobility based on five areas a person reports moving during the preceding 4 weeks; to other rooms of the house, to an area outside their home, to places in the local area, to outside the local area but in their town, to places outside their town. Four values for each of the five areas were obtained and summed giving a total for each area, accounting for frequency of movement and use of assistance from equipment or other persons.[2] Summed values for each area provide a total LSA score, ranging between 0 (totally bed-bound) to 120 (travelled out of town every day without assistance).

The 12-item GHQ, a screening tool used to quantify the severity of psychological distress experienced by an individual during the past few weeks, was used to measure well-being.[3] The GHQ

is. Each item on the scale is scored using 4 responses ranging from “better than usual” to “worse than usual”. A Likert scoring scale (0-1-2-3) was chosen over the GHQ scoring scale (0-0-1-1) as the Likert scoring scale offers a greater degree of discriminatory power, offering greater sensitivity to changes in well-being following the LWAHT programme.[4] Completion of the GHQ and totalling the scores for each of the 12 items resulted in a total GHQ score ranging from 0 to 36. Higher scores indicate worse health.

Goal Attainment Scale (GAS) quantifies the extent to which a patient’s goals are met following rehabilitation. [5,6] While goals set are individualised and differ, standardised scores allow comparison of outcomes. GAS involves establishing with the patient realistic expectations for what goals are likely to be achieved, and specific criteria for defining a successful outcome for each goal. Goal achievement is rated on a 5-point scale from -2 (much worse than expected) to +2 (much better than expected) with 0 meaning the goal was reached to the expected level. Goals were weighted to account for both the difficulty of the goal and the importance of the goal to the patient. GAS T-scores were calculated as the outcome measure using an Excel spreadsheet provided by Turner-Stokes et al.[7]

1. May D, Nayak U, Isaacs B. The life-space diary: a measure of mobility in old people at home. *International rehabilitation medicine* 1985; **7**(4): 182-6.
2. Baker PS, Bodner EV, Allman RM. Measuring life-space mobility in community-dwelling older adults. *Journal of the American Geriatrics Society* 2003; **51**(11): 1610-4.
3. Goldberg D, Williams P. A user’s guide to the General Health Questionnaire. nferNelson. *Windsor, UK* 1988.
4. Hankins M. The reliability of the twelve-item general health questionnaire (GHQ-12) under realistic assumptions. *BMC public health* 2008; **8**(1): 1-7.
5. Rockwood K, Joyce B, Stolee P. Use of goal attainment scaling in measuring clinically important change in cognitive rehabilitation patients. *Journal of clinical epidemiology* 1997; **50**(5): 581-8.
6. Stolee P, Zaza C, Pedlar A, Myers AM. Clinical experience with goal attainment scaling in geriatric care. *Journal of Aging and Health* 1999; **11**(1): 96-124.
7. Turner-Stokes L. Goal attainment scaling (GAS) in rehabilitation: a practical guide. *Clinical rehabilitation* 2009; **23**(4): 362-70.
